# Supplementary material for: A clinically validated framework for auditing AI chatbot behavior in mental health interactions
Source: Nat Med. 2026 Aug 7;32(10):3730–40. doi: 10.1038/s41591-026-04577-2 (PMC13668686; doi:10.1038/s41591-026-04577-2)
Supplement: Supplementary file 2 [file 41591_2026_4577_MOESM2_ESM.pdf]

nature portfolio

Corresponding author(s): Veith Weilnhammer & Matthew M. Nour

Last updated by author(s): Jul 6, 2026

Reporting Summary

Nature Portfolio wishes to improve the reproducibility of the work that we publish. This form provides structure for consistency and transparency in reporting. For further information on Nature Portfolio policies, see our Editorial Policies and the Editorial Policy Checklist.

Statistics

For all statistical analyses, confirm that the following items are present in the figure legend, table legend, main text, or Methods section.

n/a	Confirmed	
☐	☒	The exact sample size (*n*) for each experimental group/condition, given as a discrete number and unit of measurement
☐	☒	A statement on whether measurements were taken from distinct samples or whether the same sample was measured repeatedly
☐	☒	The statistical test(s) used AND whether they are one- or two-sided<br>*Only common tests should be described solely by name; describe more complex techniques in the Methods section.*
☐	☒	A description of all covariates tested
☐	☒	A description of any assumptions or corrections, such as tests of normality and adjustment for multiple comparisons
☐	☒	A full description of the statistical parameters including central tendency (e.g. means) or other basic estimates (e.g. regression coefficient) AND variation (e.g. standard deviation) or associated estimates of uncertainty (e.g. confidence intervals)
☐	☒	For null hypothesis testing, the test statistic (e.g. *F*, *t*, *r*) with confidence intervals, effect sizes, degrees of freedom and *P* value noted<br>*Give P values as exact values whenever suitable.*
☒	☐	For Bayesian analysis, information on the choice of priors and Markov chain Monte Carlo settings
☐	☒	For hierarchical and complex designs, identification of the appropriate level for tests and full reporting of outcomes
☐	☒	Estimates of effect sizes (e.g. Cohen's *d*, Pearson's *r*), indicating how they were calculated

Our web collection on statistics for biologists contains articles on many of the points above.

Software and code

Policy information about availability of computer code

Data collection	Simulated conversations were generated with the Petri auditing agent on the Inspect AI framework under Python 3.10, with model calls routed via OpenRouter.
Data analysis	Analyses were run in R 4.6.1 with Quarto 1.9.38. Mixed-effects and ordinal models used lme4 2.0.1, lmerTest 3.2.1 and ordinal 2025.12.29; contrasts and inference used car 3.1.5, emmeans 2.0.3 and psych 2.6.5; data handling and figures used tidyverse 2.0.0, ggplot2 4.0.3 and viridis 0.6.5. Analysis code: https://github.com/veithweilnhammer/sim-vail.

For manuscripts utilizing custom algorithms or software that are central to the research but not yet described in published literature, software must be made available to editors and reviewers. We strongly encourage code deposition in a community repository (e.g. GitHub). See the Nature Portfolio guidelines for submitting code & software for further information.

Data

Policy information about availability of data

All manuscripts must include a data availability statement. This statement should provide the following information, where applicable:

- Accession codes, unique identifiers, or web links for publicly available datasets
- A description of any restrictions on data availability
- For clinical datasets or third party data, please ensure that the statement adheres to our policy

Data supporting this study are publicly available without access restrictions in the SIM-VAIL repository (https://github.com/veithweilnhammer/sim-vail), archived at

Zenodo (DOI: 10.5281/zenodo.21208170), under the MIT License. The release includes synthetic conversation transcripts with turn- and conversation-level safety scores, control simulations, and documentation. The synthetic transcripts contain no human data.

Research involving human participants, their data, or biological material

Policy information about studies with human participants or human data. See also policy information about sex, gender (identity/presentation), and sexual orientation and race, ethnicity and racism.

Reporting on sex and gender	N/A. SIM-VAIL reports only simulated data. The simulated user profiles were not designed to represent sex- or gender-specific effects.
Reporting on race, ethnicity, or other socially relevant groupings	N/A. SIM-VAIL reports only simulated data. The simulated user profiles were not designed to represent race, ethnicity or other socially relevant groupings
Population characteristics	N/A
Recruitment	N/A
Ethics oversight	N/A

Note that full information on the approval of the study protocol must also be provided in the manuscript.

Field-specific reporting

Please select the one below that is the best fit for your research. If you are not sure, read the appropriate sections before making your selection.

☐ Life sciences ☒ Behavioural & social sciences ☐ Ecological, evolutionary & environmental sciences

For a reference copy of the document with all sections, see nature.com/documents/nr-reporting-summary-flat.pdf

Behavioural & social sciences study design

All studies must disclose on these points even when the disclosure is negative.

Study description	Quantitative computational (in-silico) simulation study: 810 simulated multi-turn conversations (30 user profiles = 5 vulnerabilities × 6 intents, across 9 AI chatbots, in 3 replicates), scored for concerning behavior by automated LLM judges and validated against blinded clinician annotations.
Research sample	810 simulated conversations across 9 chatbot models.
Sampling strategy	No sample-size calculation was performed; the design is a full factorial (30 profiles × 9 models × 3 replicates = 810), sized to cover every profile × model combination with replication.
Data collection	Conversations were generated automatically via the Petri/Inspect harness through OpenRouter, with no human experimenter in the loop.
Timing	Simulated conversations were generated in October–November 2025. Additional analyses were performed in April 2026 (R1).
Data exclusions	No data were excluded from the analyses.
Non-participation	N/A
Randomization	Conditions were assigned by full factorial design, not random allocation of participants; profile × model × replicate cells were fully crossed, with replicate variation arising from model sampling.

Reporting for specific materials, systems and methods

We require information from authors about some types of materials, experimental systems and methods used in many studies. Here, indicate whether each material, system or method listed is relevant to your study. If you are not sure if a list item applies to your research, read the appropriate section before selecting a response.

Materials & experimental systems

n/a	Involved in the study
☒	☐ Antibodies
☒	☐ Eukaryotic cell lines
☒	☐ Palaeontology and archaeology
☒	☐ Animals and other organisms
☒	☐ Clinical data
☒	☐ Dual use research of concern
☒	☐ Plants

Methods

n/a	Involved in the study
☒	☐ ChIP-seq
☒	☐ Flow cytometry
☒	☐ MRI-based neuroimaging

Plants

Seed stocks	*Report on the source of all seed stocks or other plant material used. If applicable, state the seed stock centre and catalogue number. If plant specimens were collected from the field, describe the collection location, date and sampling procedures.*
Novel plant genotypes	*Describe the methods by which all novel plant genotypes were produced. This includes those generated by transgenic approaches, gene editing, chemical/radiation-based mutagenesis and hybridization. For transgenic lines, describe the transformation method, the number of independent lines analyzed and the generation upon which experiments were performed. For gene-edited lines, describe the editor used, the endogenous sequence targeted for editing, the targeting guide RNA sequence (if applicable) and how the editor was applied.*
Authentication	*Describe any authentication procedures for each seed stock used or novel genotype generated. Describe any experiments used to assess the effect of a mutation and, where applicable, how potential secondary effects (e.g. second site T-DNA insertions, mosiacism, off-target gene editing) were examined.*
